# Brain-based correlates of depression and traumatic brain injury: a systematic review of structural and functional magnetic resonance imaging studies

**DOI:** 10.3389/fnimg.2024.1465612

**Published:** 2024-11-05

**Authors:** Vanessa A. Baltazar, Ilya Demchenko, Vanessa K. Tassone, Rachel L. Sousa-Ho, Tom A. Schweizer, Venkat Bhat

**Affiliations:** ^1^Interventional Psychiatry Program, St. Michael's Hospital, Toronto, ON, Canada; ^2^Temerty Faculty of Medicine, Institute of Medical Science, University of Toronto, Toronto, ON, Canada; ^3^Neuroscience Research Program, St. Michael's Hospital, Toronto, ON, Canada; ^4^Division of Neurosurgery, Department of Surgery, Temerty Faculty of Medicine, University of Toronto, Toronto, ON, Canada; ^5^Department of Psychiatry, Temerty Faculty of Medicine, University of Toronto, Toronto, ON, Canada

**Keywords:** depressive disorder, brain injuries, magnetic resonance imaging, neural pathways, neuroimaging, systematic review, traumatic brain injury

## Abstract

**Introduction:**

Depression is prevalent after traumatic brain injury (TBI). However, there is a lack of understanding of the brain-based correlates of depression post-TBI. This systematic review aimed to synthesize findings of structural and functional magnetic resonance imaging (MRI) studies to identify consistently reported neural correlates of depression post-TBI.

**Methods:**

A search for relevant published studies was conducted through OVID (MEDLINE, APA PsycINFO, and Embase), with an end date of August 3rd, 2023. Fourteen published studies were included in this review.

**Results:**

TBI patients with depression exhibited distinct changes in diffusion- based white matter fractional anisotropy, with the direction of change depending on the acuteness or chronicity of TBI. Decreased functional connectivity (FC) of the salience and default mode networks was prominent alongside the decreased volume of gray matter within the insular, dorsomedial prefrontal, and ventromedial prefrontal cortices. Seven studies reported the correlation between observed neuroimaging and depression outcomes. Of these studies, 42% indicated that FC of the bilateral medial temporal lobe subregions was correlated with depression outcomes in TBI.

**Discussion:**

This systematic review summarizes existing neuroimaging evidence and reports brain regions that can be leveraged as potential treatment targets in future studies examining depression post-TBI.

## 1 Introduction

Depression affects 47.5% of the general population and continues to present an ever-increasing economic burden (Greenberg et al., 2021). Evidence suggests that there is a high prevalence of depression associated with traumatic brain injury (TBI) (Singh et al., 2018). Specifically, 17% of TBI patients are diagnosed with major depressive disorder (MDD) 1 year after injury (Scholten et al., 2016). In addition, 43% of TBI patients are diagnosed with depressive disorders over time (Scholten et al., 2016). The treatment of depression is complex, as the disorder itself is highly heterogeneous, with approximately 30% of cases being resistant to conventional antidepressant treatment (Voineskos et al., 2020). In addition, meta-analyses show that standard antidepressant treatment has no significant effect on depressive symptoms post-TBI (Kreitzer et al., 2019; Slowinski et al., 2019). This observation implies that there are potentially distinct neural mechanisms responsible for depressive symptomatology in TBI compared to MDD alone. A key step forward in overcoming this poor treatment response involves understanding the pathophysiology of depression in TBI. Conceptualizing the disorder as a dysfunction of brain circuitry may direct the field of psychiatry to alternative available treatments, such as neuromodulation or psychotherapy, which may better target depression post-TBI (Jahan and Tanev, 2023).

Depression is characterized by structural and functional changes in the brain. For white matter (WM), recent papers have associated depression with decreased integrity of WM tracts, including the corpus callosum (CC), fronto-occipital fasciculus, and cingulum (van Velzen et al., 2020). For gray matter (GM), brain regions like the parahippocampal gyrus, amygdala, and insula exhibit volumetric reductions (Romeo et al., 2024). In addition, structural changes in GM and WM that are associated with depression can contribute to functional changes in key brain networks. Depression is linked to disrupted functional connectivity within the default mode network (DMN), central executive network (CEN), and salience network (SN) (Dai et al., 2019; Demchenko et al., 2022), as well as between the nodes of these different networks. For example, in depression, there is a characteristic inappropriate increase in functional connectivity between the SN and DMN, whereas the DMN shows decreased functional connectivity with CEN (Dai et al., 2019).

Structural and functional neuroimaging is a valuable tool in the toolkit, which provides insights into the neurological underpinnings of depression post-TBI. However, there is a need to further understand the correlation between neuroimaging and depression outcomes in TBI patients. An emphasis on the investigation of depression post-TBI through correlational studies may aid in identifying brain regions with altered structure or function in TBI that may contribute to depressive pathophysiology. For example, volumetric studies suggest that several changes in GM observed in TBI coincide with changes seen in depression. These changes include increased volume of GM in the cingulate and paracingulate cortices and the right caudate nucleus, and decreased volume in the frontal and temporal lobes, hippocampus, and right thalamus (Mavroudis et al., 2022; Gray et al., 2020). However, there is also evidence of no significant association between reduced GM volume in limbic areas and depression scores in TBI cohorts (Luo et al., 2023). In TBI, the prolonged activation of microglia and their production of pro-inflammatory cytokines can result in neuronal damage, synaptic loss, and, in some cases, death of neurons—all these processes may lead to GM volume reduction in affected brain regions (Niu et al., 2020). With this observation, there are proposed hypotheses on how prolonged neuroinflammation is associated with depression-like symptoms, such as anhedonia, anorexia, and cognitive disturbances (Maes et al., 2009). However, more research is needed to clarify the association between neuroinflammation post-TBI and depressive symptoms. There is also a lack of research on the possible role of WM abnormalities in depression post-TBI. Changes in the integrity of WM tracts, such as the inferior longitudinal fasciculus (ILF) and anterior part of the cingulum, are associated not only with depression but also TBI (Zheng et al., 2019). However, there is no evidence that suggests that impaired integrity of these tracts in TBI is associated with the subsequent development of depression (Wallace et al., 2018).

In addition, recent studies have shown a stronger correlation between functional neuroimaging outcomes and observed depression outcomes in TBI patients compared to volumetry-based outcomes (Luo et al., 2023). Functional neuroimaging outcomes may thus further delineate the pathology of depressive symptoms post-TBI beyond what is currently known based on volumetric studies. Functional connectivity (FC) studies of depression show dysfunction of the SN and DMN (Fischer et al., 2016). However, there is evidence suggesting that the disruption of these networks is associated with cognitive impairment but not necessarily with depressive symptoms post-TBI (Lancaster et al., 2019). Likewise, studies reporting blood-oxygen-level-dependent (BOLD) and cerebral blood flow (CBF) outcomes have provided insight into the possible involvement of the insula in TBI (Lu et al., 2020; Meier et al., 2015), but a link to depression post-TBI is yet to be established.

The purpose of this systematic review is to synthesize findings of magnetic resonance imaging (MRI) studies that reported changes in structural (GM volume, WM diffusion) and functional (BOLD, FC) outcomes in patients with depression post-TBI. By identifying potential neuroimaging-based biomarkers associated with depression post-TBI, future therapeutic and diagnostic approaches can be refined. In addition, this review will add to the work of previous systematic reviews that have provided a comprehensive outline of structural MRI studies on TBI and depression, and it will also establish a framework for functional MRI (fMRI)-based correlates of depression post-TBI (Maller et al., 2010; Medaglia, 2017). To our knowledge, there is currently no systematic review that examines depression post-TBI with a combined focus on structural and functional MRI outcomes.

## 2 Materials and methods

This systematic review followed the Preferred Reporting Items for Systematic Reviews and Meta-Analyses (PRISMA) guidelines (Page et al., 2021).

### 2.1 Search strategy

A comprehensive search for structural and functional MRI studies involving patients with depression in TBI was conducted through OVID (MEDLINE, APA PsycINFO, Embase), with an end date of August 3rd, 2023. Four independent searches corresponding to different types of studies (i.e., functional activation, FC, anatomical WM connectivity, anatomical GM volume) were performed, and full search strategies are provided in Appendix A. Briefly, the search terms included, but were not limited to, traumatic brain injury^*^, TBI^*^, depressive disorder^*^, magnetic resonance imag^*^, BOLD-contrast imag^*^, and diffusion magnetic resonance imag^*^.

### 2.2 Inclusion and exclusion criteria

Two authors (V.A.B and R.L.S) independently conducted the search and screened studies for inclusion. Discrepancies were discussed and resolved by a third party (V.K.T). Studies were included if data were collected using an MRI method (i.e., structural MRI, fMRI, perfusion-weighted MRI, diffusion-weighted MRI). Studies were only included if they reported on WM, GM, FC and BOLD outcomes and were peer-reviewed. In addition, studies were included if participants with TBI were assessed for the presence of depressive symptoms (i.e., diagnosed or measured using a validated scale) and were antidepressant-free at baseline in both interventional and non-interventional studies. Additionally, studies were screened for the following criteria: (1) participants aged 18–65 (if the age range was not provided and the mean age was between 18 and 65, the study was excluded), and (2) the study was published in English. Studies were excluded if some or all patients met the criteria for comorbid psychiatric or neurological disorders, except for anxiety due to its high co-occurrence with depression, or if patients had a depression diagnosis prior to TBI. Studies with patients outside of the 18-65 age range were excluded since geriatric and pediatric TBI have their own distinct prognosis models compared to adult TBI (Gardner et al., 2018; Araki et al., 2017). Studies were also excluded if they investigated post-stroke depression or postpartum depression. There was no restriction on the year of publication or the sex of participants. Review articles and meta-analyses were excluded.

### 2.3 Variable extraction

Data extraction was done by authors V.A.B and V.K.T. The extracted variables included participant characteristics (i.e., mean age, sex, TBI diagnosis criteria, time after injury, depression scale, comorbid anxiety, history of treatment, and number of female participants), year of publication, and country where the study was conducted. Information on the study inclusion criteria was extracted to understand how TBI and depression were diagnosed and/or assessed. Details concerning structural and functional MRI methodology were also extracted (i.e., MRI acquisition parameters and tasks), along with results pertaining to changes in GM, WM, FC, BOLD/hemodynamic response, and the correlation between reported clinical and neuroimaging outcomes.

### 2.4 Quality assessment

Quality Assessment was done by authors V.A.B and R.L.S. Published studies included in the review were assessed for quality using the JBI (formerly Joanna Briggs Institute) Checklist for Case Control Studies (Aromataris and Munn, 2020).

## 3 Results

**Figure 1:**
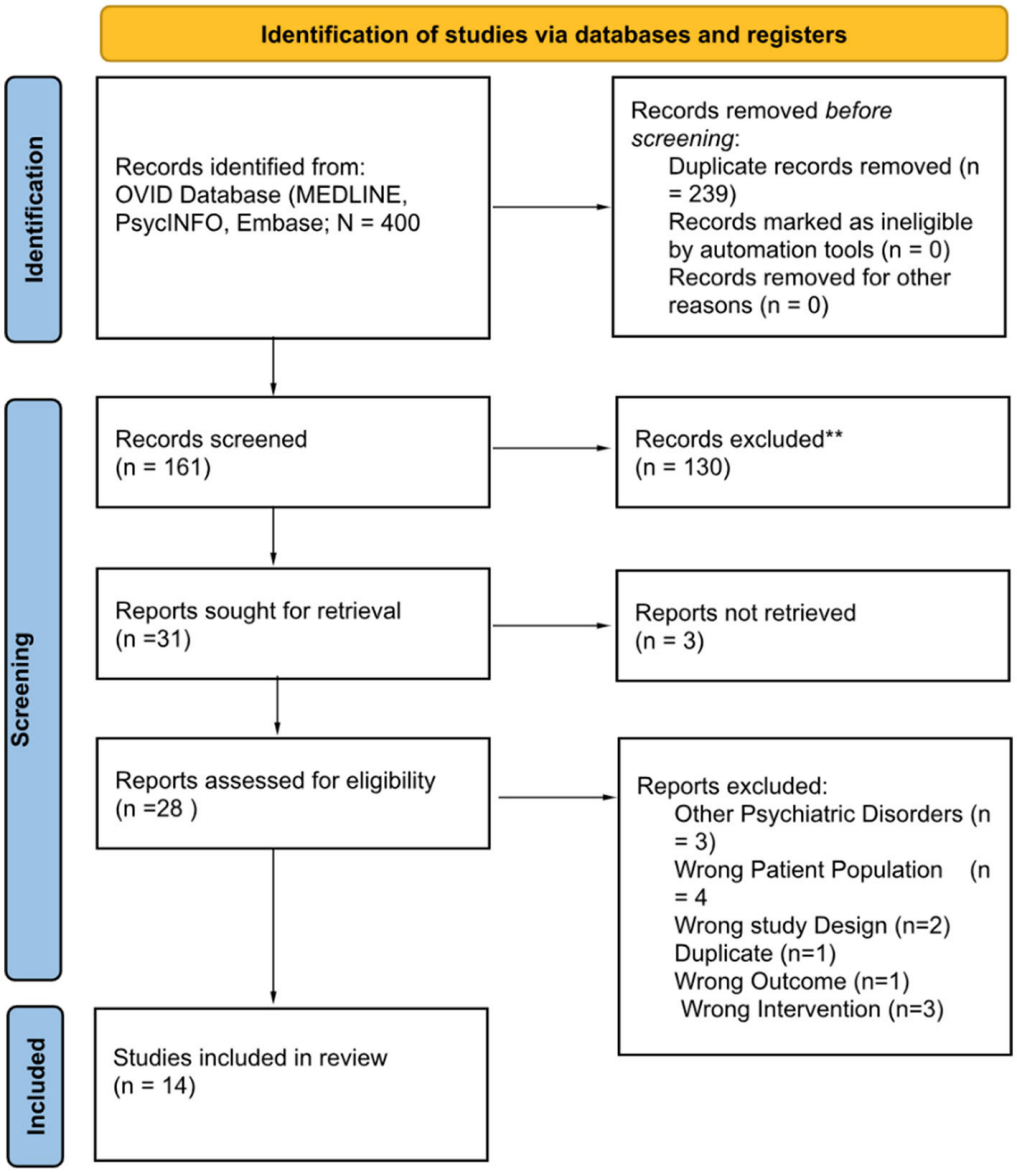
PRISMA flow diagram of studies screened and assessed for the systematic review.

After the initial search, duplicates were removed, and studies were screened at the first level (i.e., titles and abstracts). Of the 400 original reports identified by the database searches, 28 full texts were assessed for eligibility at the second level. In total, 14 published studies were included in the review (Figure 1).

### 3.1 Quality assessment

The total score of “Yes” that each study received from the 10-item checklist was as follows: Spirou et al. (2019), Raikes et al. (2018), Matthews et al. (2012), Jolly et al. (2019), Huang et al. (2022), Maller et al. (2014a,b), Simos et al. (2023), and Luo et al. (2023) received a 10/10; McCuddy et al. (2018) received a 9/10; Papadaki et al. (2021) received an 8/10; Choi and Jang, 2021, Jang et al. (2016), and Gao et al. (2022) received a 7/10. The results of the quality assessment are provided and summarized in Appendix B. Based on the results of the quality assessment, all papers were considered equal in quality and were appraised equally when being reported in the review.

### 3.2 Study characteristics

**Figure 2:**
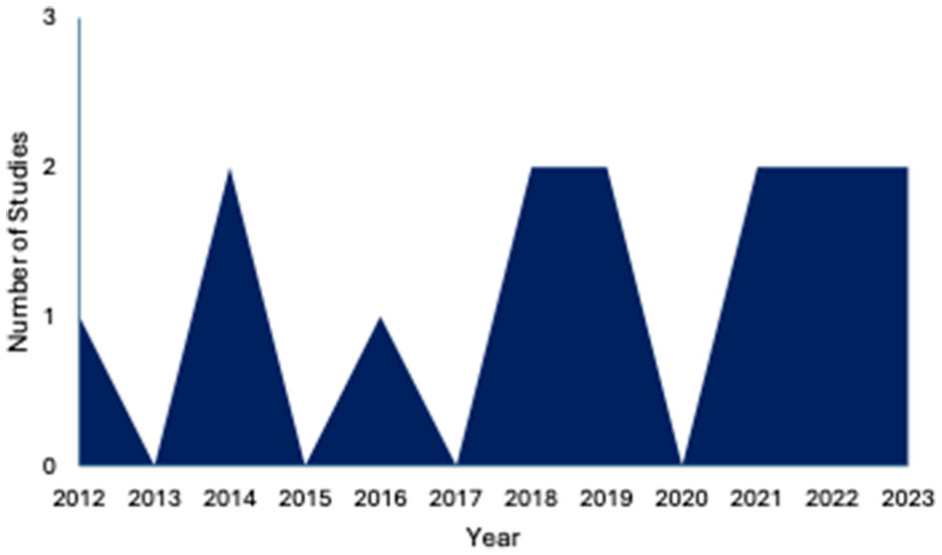
Number of published studies per year, *n* = 14.

**Figure 3:**
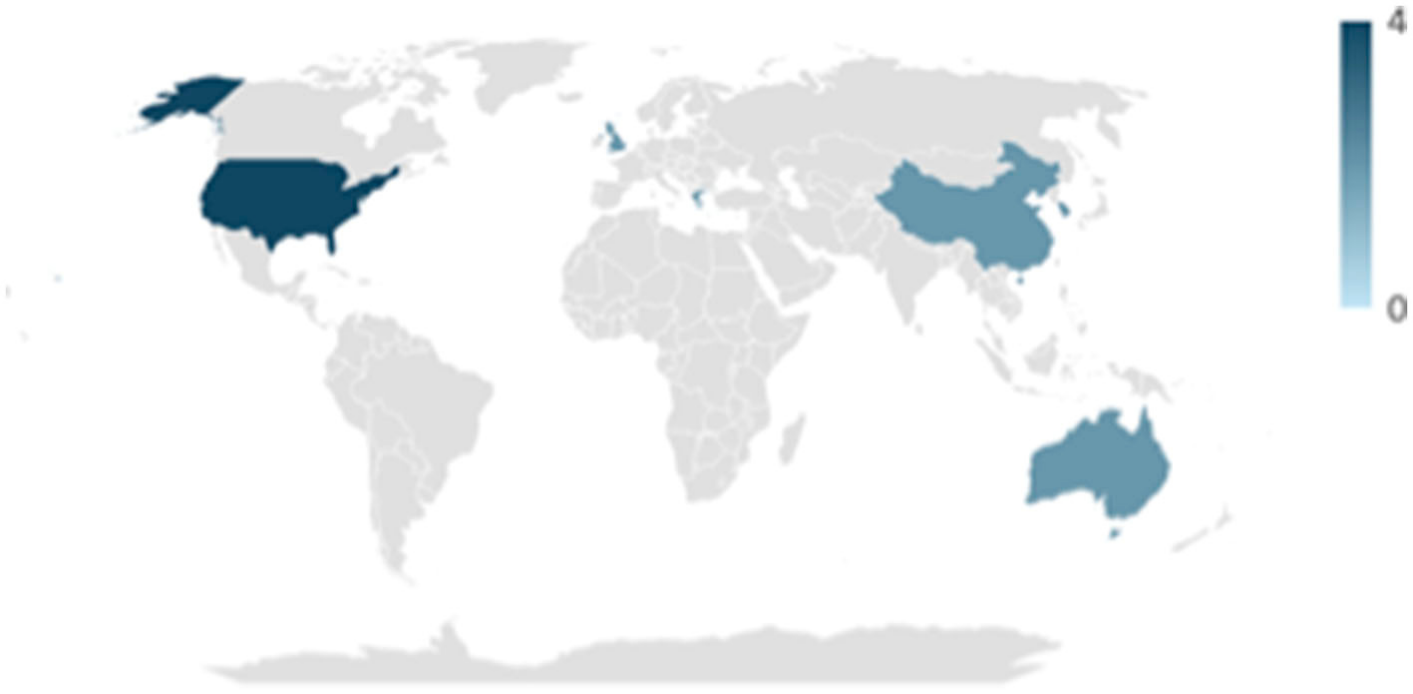
Number of published studies per country, *n* = 14.

Studies were published between 2012 and 2022, with nine studies being published from 2015 onwards (Figure 2). Four studies were conducted in the United States (McCuddy et al., 2018; Spirou et al., 2019; Raikes et al., 2018; Matthews et al., 2012), and other studies were conducted in Greece (Papadaki et al., 2021; Simos et al., 2023), China (Huang et al., 2022; Gao et al., 2022), the United Kingdom (Jolly et al., 2019; Luo et al., 2023), Australia (Maller et al., 2014a,b), and the Republic of Korea (Choi and Jang, 2021; Jang et al., 2016) (Figure 3).

### 3.3 Demographic and clinical characteristics of participants

The mean (standard deviation [SD]) age of participants ranged from 20.29 (1.31) to 45.40 (16.90) across TBI groups. Approximately 15.66% of the participants were female. Time after TBI ranged from 1 day to 10 years. TBI was most often assessed with the Glasgow Coma Scale (GCS) (Papadaki et al., 2021; Choi and Jang, 2021; Jang et al., 2016; Maller et al., 2014a,b; Simos et al., 2023; Luo et al., 2023), whereas depression was predominantly assessed with the Beck Depression Inventory (BDI)-II (Choi and Jang, 2021; Raikes et al., 2018; Matthews et al., 2012; Jolly et al., 2019; Jang et al., 2016; Luo et al., 2023). Table 1 summarizes the demographic and clinical characteristics of participants from each included study.

**Table 1:** Participant characteristics for included studies.

**References**	**TBI group demographics**	**Mean age (*SD*), years**	**Female *N* (%)**	**Diagnostic criteria**	**TBI Scale score for participant inclusion**	**Depression Scale**	**Mean depression severity (SD) at baseline**	**Healthy control demographics**	**Mean age (*SD*), years**	**Female *N* (%)**
	***N***							***N***		
Matthews et al. (2012)	46	LOC = 29.12 (6.07); AOC = 26.59 (4.92)	0	Traumatic Brain Injury Screen, TBI questionnaire	N/A	BDI-II	LOC: 20.00 (11.77), AOC:12.83 (10.27)	0	N/A	N/A
Maller et al. (2014a)	26	TBI-MDD: 48.00 (9.92), TBI-no-MD: 33.08 (12.69)	10 (38.46)	GCS	GCS <13-14	MADRS	TBI-MD:28.77 (7.68), TBI-no-MD: 2.25 (2.38)	28	38.35 (13.00)	14 (60.87)
Maller et al. (2014b)	26	TBI-MDD: 48.00 (9.92), TBI-no-MD: 33.08 (12.69)	10 (38.46)	GCS	GCS <13-14	MADRS	TBI-MD:28.77 (7.68), TBI-no-MD: 2.25 (2.38)	28	38.35 (13.00)	14 (60.87)
Jang et al. (2016)	1	63	1 (100)	GCS	GCS = 15	BDI-II,PHQ	BDI-II= 42, PHQ=24	10	62.30 (3.10)	N/A
McCuddy et al. (2018)	43	20.29 (1.31)	9 (20.93)	Diagnosed Independent of the Study	N/A	HAM-D 21-Item	N/A	51	20.26 (1.44)	16 (31.37)
Raikes et al. (2018)	34	24.40 (7.40)	21 (61.76)	GCS	GCS <13–15	BDI-II	9.60 (8.10)	18	23.20 (3.40)	9 (50)
Jolly et al. (2019)	12	36.90 (9.90)	4 (33.33)	Mayo Criteria (Malec et al., 2007)	N/A	BDI	N/A	58	35.90 (6.90) 37.60 (9.10)	4 (7.27%)
Spirou et al. (2019)	16	41.56 (9.99)	7 (43.75)	Independent of the Study	N/A	CMDI	N/A	N/A	N/A	N/A
Choi and Jang (2021)	1	51	0	GCS	GCS <3	BDI-II	41	N/A	N/A	N/A
Papadaki et al. (2021)	32	45.40 (16.90)	7 (21.88)	GCS	GCS > 13	CESD	13.71 (9.30)	31	44.40 (17.0)	19 (61.29)
Gao et al. (2022)	30	35.30 (10.33)	11 (36.67)	GCS	N/A	HADS	7.63 (5.83)	20	32.95 (9.25)	6 (30)
Huang et al. (2022)	42	34.17 (11.30)	19 (45.24)	American Congress of Rehabilitation Medicine for mTBI	At least one of the diagnostic criteria	SRDS	46.67(11.99)	37	35.46 (10.72)	18 (48.65)
Luo et al. (2023)	79	38 (16.13)	22 (28)	GCS	Severe TBI= GCS <8; Moderate TBI= 8 ≤ GCS ≤ 12; Mild TBI= GCS > 12	BDI-II	9.84 (8.67)	0	N/A	N/A
Simos et al. (2023)	37	40.33 (17.40)	N/A (16)	GCS	N/A	CESD	12.30 (8.8)	39	41.73(15.6)	N/A (28)

AOC, alteration of consciousness; BDI, Beck Depression Inventory; BDI-II, Beck Depression Inventory-II; CESD, Center for Epidemiological Studies Depression Scale; CMDI, Chicago Multiscale Depression Inventory; DSM-5, Diagnostic and Statistical Manual of Mental Disorders, fifth edition; DSM-IV, Diagnostic and Statistical Manual of Mental Disorders, fourth edition; DSM-IV-TR, text revision of Diagnostic and Statistical Manual of Mental Disorders, fourth edition; GCS, Glasgow Coma Scale; HADS, Hospital Anxiety and Depression Scale; HAM-D, Hamilton Rating Scale for Depression; HAMD-17-Mod, 17-item Hamilton Rating Scale for Depression omitting sleep and weight loss items; HC, healthy control subjects; LOC, loss of consciousness; MADRS, Montgomery-Åsberg Depression Rating Scale; MDD, major depressive disorder; N, sample size; N/A, data not given in the article; SCID, Structured Clinical Interview for DSM Disorders; SD, standard deviation; SRDS, Zung Self-Rated Depression Scale; TBI, traumatic brain injury.

### 3.4 Neuroimaging techniques used

**Figure 4:**
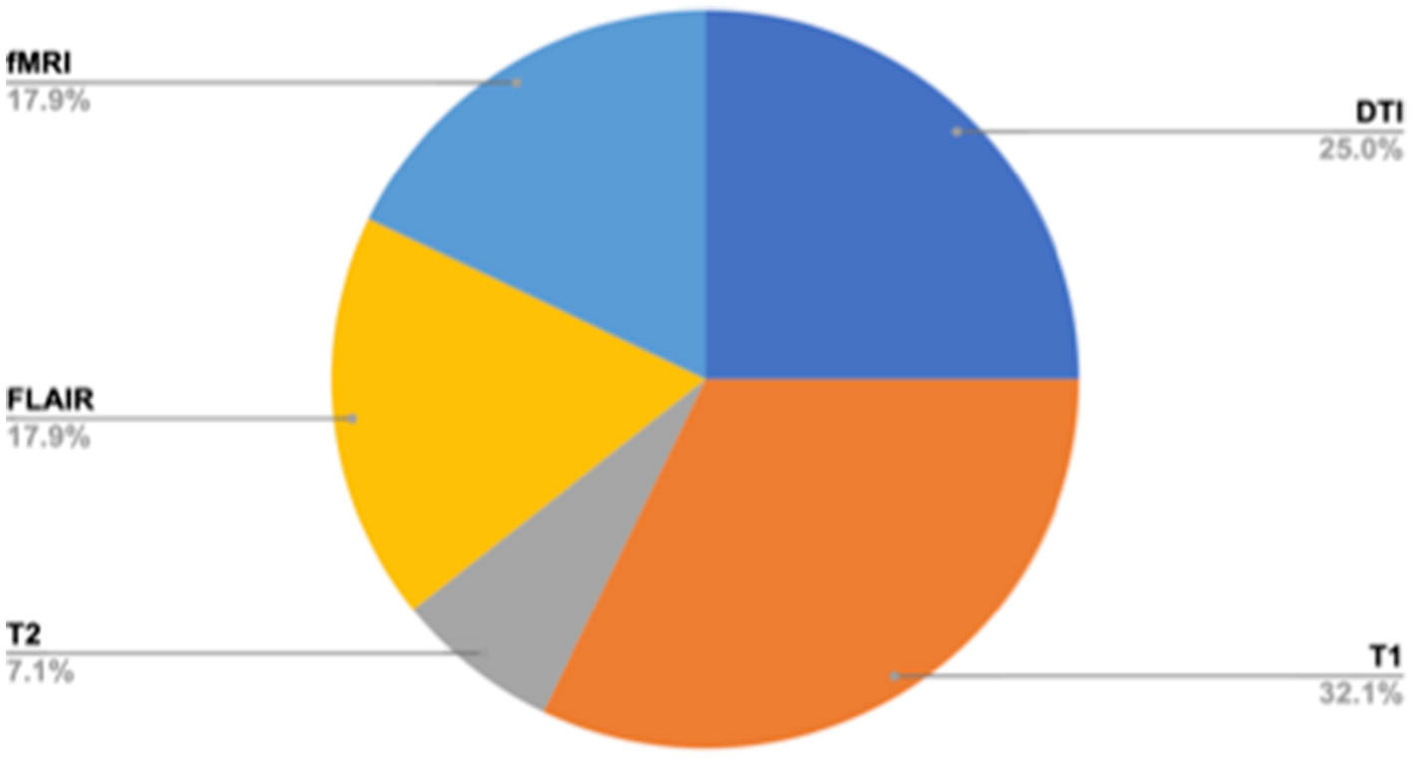
Percentage of published studies per neuroimaging modality, *n* = 14.

Across the published studies included in the review, the most commonly acquired MRI sequences included the T1-weighted scan in nine studies (McCuddy et al., 2018; Papadaki et al., 2021; Spirou et al., 2019; Jolly et al., 2019; Huang et al., 2022; Gao et al., 2022; Maller et al., 2014a,b; Luo et al., 2023) and diffusion tensor imaging (DTI) scan in seven studies (Choi and Jang, 2021; Raikes et al., 2018; Matthews et al., 2012; Jolly et al., 2019; Higgins et al., 2019; Jang et al., 2016; Maller et al., 2014a). Two studies acquired a T2-weighted scan (Papadaki et al., 2021; Jang et al., 2016), five acquired a fluid-attenuated inversion recovery (FLAIR) scan (Jolly et al., 2019; Huang et al., 2022; Gao et al., 2022; Maller et al., 2014a,b), and four acquired an fMRI scan, either a task-based (Spirou et al., 2019) or resting-state (McCuddy et al., 2018; Luo et al., 2023; Simos et al., 2023) (Figure 4). Table 2 summarizes the MRI acquisition sequences and metrics used in each study, along with a summary of their results.

**Table 2:** Neuroimaging methods and findings for published studies included in the review.

**References**	**MRI aqusition**	**Task(s) performed**	**Gray/white structural alterations**	**Functional connectivity and hemodynamic outcomes**
Matthews et al. (2012)	DTI	Resting State	•14 regions within the brainstem, corpus callosum, cingulate gyrus, inferior and superior longitudinal fasciculus, inferior frontal occipital fasciculus, anterior limb of the internal capsule, anterior thalamic radiation, and anterior corona radiata, where FA was significantly lower in the LOC compared to the AOC group	•N/A
Maller et al. (2014a)	FLAIR, T1w, DTI	Resting State	•Reduced FA in the TBI sample was not statistically significantly (P 1/4 0.10, corrected) in TBI and control comparisons •RD was greater in the TBI sample at the P 1/4 0.05 level (corrected) across widespread areas including the CC, bilateral PFC, superior longitudinal fasciculi, and right internal capsule •Significant large cluster for reduced volume in the TBI sample for the left hemisphere located in the left inferior parietal region (including the supramarginal and angular gyri, extending to the lateral occipital) and for volume in the right insular region extending to the pars triangularis; right hippocampal volume to be significantly larger in the controls (F(1,47) = 4.781, p = 0.034)	•N/A
Maller et al. (2014b)	T1w, FLAIR	Resting State	•TBI vs Control: significant large cluster for reduced volume in the TBI sample for the left hemisphere located in the left inferior parietal region (including the supramarginal and angular gyri, extending to the lateral occipital) and for volume in the right insular region extending to the pars triangularis; right hippocampal volume to be significantly larger in the controls [F_(1, 47)_ = 4.781, *p* = 0.034] •TBI–MDD vs. TBI-no-MDD: decrease among the TBI–MDD sample in the left hemisphere with two large significant clusters in the inferior temporal and inferior parietal regions •Within the right hemisphere, there was one significant cluster in the lingual region; the following regions were significantly larger in those with TBI-no-MDD: left accumbens [F_(3, 12)_ = 4.060, *p* = 0.033], right accumbens [F_(3, 12)_ = 4.207, *p* = 0.030] TBI–MDD vs MDD-no-TBI: one large cluster was significant (right hemisphere) in the inferior temporal region (larger in the MDD-no-TBI group)	•N/A
Jang et al. (2016)	DTI	Resting State	•Ventrolateral prefronto- and orbitofronto-thalamic tracts were well reconstructed in both hemispheres •Partial tearing of the dorsolateral prefronto-thalamic tract was observed in the right hemisphere and thinning in the left hemisphere.	•N/A
McCuddy et al. (2018)	fMRI, T1w	Resting State	•N/A	•Significant difference in connectivity of the meta-analytically derived emotional processing network at T3 relative to controls (χ2 = 8.95, p = 0.0028), •Pairwise longitudinal comparisons in concussed athletes demonstrated that connectivity in the emotion processing network was significantly different at T3 relative to T1 (χ2 = 9.66, p = 0.0019)
Raikes et al. (2018)	DTI	Resting State	•No statistically significant differences observed between the healthy control participants and those with a history of mTBI for any of the diffusion metrics at the whole brain level (a priori α = 0.05)	•N/A
Jolly et al. (2019)	DTI		•Significantly reduced FA in TBI-MDD patients (*p* = 0.007) and TBI-no-MDD patients (*p* = 0.001) compared to controls for the cingulum. significantly reduced FA in TBI-MDD patients compared to controls (*p* = 0.03) for the uncinate fasciculus	•N/A
Spirou et al. (2019)	fMRI	Card Game	•No difference in GM volume between groups •No significant volumetric differences in the caudate, nucleus accumbens, and putamen bilaterally in high vs. low depression	•With CMDI-Mood as a covariate, observed widespread clusters of activation that included the VMPFC and the striatum (*P* < 0.05) in response to positive outcome presentation •No activation in any brain regions in association with punishing outcomes
Choi and Jang (2021)	DTI	Resting State	•Compared to 3-month DTT, the right DLPTT, VLPPT, OPTT, uncinate fasciculus appeared narrow except for the left cingulum, which showed new transcallosal fibers between both anterior cingula •The FA values of all reconstructed neural tracts were lower on the 8-year DTT than on the 3-month DTT •In addition, TV values of all reconstructed neural tracts, except for the left cingulum, were lower on the 8-year DTT than on the 3-month DTT	•N/A
Papadaki et al. (2021)	fMRI, T2w	Resting State	•N/A	•Compared to controls, mTBI patients displayed reduced CBF in dlPFC, putamen, and hippocampus bilaterally and reduced CBV in right dlPFC/vmPFC and hippocampus bilaterally (p <0.004)
Gao et al. (2022)	fMRI, T1w, FLAIR,	Resting State	•TBI patients had smaller SN in both left and right hemispheres compared to HC •In TBI group, the left SN was relatively small compared to the left	•TBI patients had reduced FC in the left SN compared to healthy controls •Weakened FC between left SN and bilateral superior medial frontal gyrus extending into the right anterior cingulate cortex •Left SN showed reduced FC to the left angular gyrus extending into the left inferior parietal lobe
Huang et al. (2022)	T1w, FLAIR	Resting State	•mTBI group had a more significant number of clusters with increased FA than the HCs (corrected *p* < 0.05) •The clusters belonged to the bilateral inferior fronto-occipital fasciculus, superior longitudinal fasciculus, left uncinate fasciculus, left anterior thalamic radiation, and right inferior longitudinal fasciculus •No significant changes in MD, AD, or RD between the two groups	•N/A
Luo et al. (2023)	T1w, fMRI	Resting State	•N/A	•N/A
Simos et al. (2023)	fMRI	Resting State	•N/A	•Reduced FC in the bilateral medial pole, left anterior hippocampus, right amygdala, anterior and medial prefrontal regions and dorsal posterior cingulate cortex •Increased FC in the left precuneus and dorsal posterior cingulate

ACC, anterior cingulate cortex; AD, axial diffusivity; AOC, alterations of consciousness; BDI, beck depression inventory; BDI-2, beck depression inventory-II; BOLD, blood-oxygen-level-dependent; BP-ND: non-displaceable binding potential; CBF, cerebral blood flow; CBV, cerebral blood volume; CC, corpus callosum; CMDI-Mood, chicago multiscale depression inventroy mood subscale; dlPFC, dorsal lateral prefrontal cortex; DLPTT, dorsal lateral prefrontal thalamic tract; DTI, diffusion tensor imaging; DTT, diffusion tensor tractography; FA, functional anisotropy; FC, functional connectivity; FLAIR, fluid-attenuated inversion recovery; fMRI, functional magnetic resonance imaging; GM, gray matter; HAMD, hamilton rating scale for depression; HCs, healthy control subjects; LOC, loss of consciousness; MADRS, montgomery-åsberg depression rating scale; MD, mean diffusivity; MDD, major depressive disorder participants; mTBI, mild traumatic brain injury; N/A, data not given in the article; OPTT, orbital prefrontal thalamic tract; PCC, posterior cingulate cortex; PCF, prefrontal cortex; PFC, prefrontal cortex; RD, radial diffusivity; ROI, regions of interest; SDS, self-reporting depression scale; SN, substantia nigra; TBI, traumatic brain injury; T1, time point one; T1w, T1 weighted MRI; T2w, T2 weighted MRI; T3, time point 3; VLPTT, ventral lateral prefrontal thalamic tract; vmPFC, ventromedial prefrontal cortex.

### 3.5 White matter structural differences

Seven studies presented results for the structural differences in WM. Two case studies examined structural differences in a single TBI patient (Jang et al., 2016; Choi and Jang, 2021). Two studies made comparisons between MDD and non-MDD post-TBI patients in addition to comparisons between depression post-TBI patients and healthy control (HC) groups (Jolly et al., 2019; Maller et al., 2014a). Two studies only made comparisons between depression post-TBI patients and HC groups (Huang et al., 2022; Raikes et al., 2018). Lastly, a study by Matthews et al. (2012) only examined structural differences between depression post-TBI patients who experiences either loss of consciousness (LOC) or alterations in consciousness (AOC).

**Figure 5:**
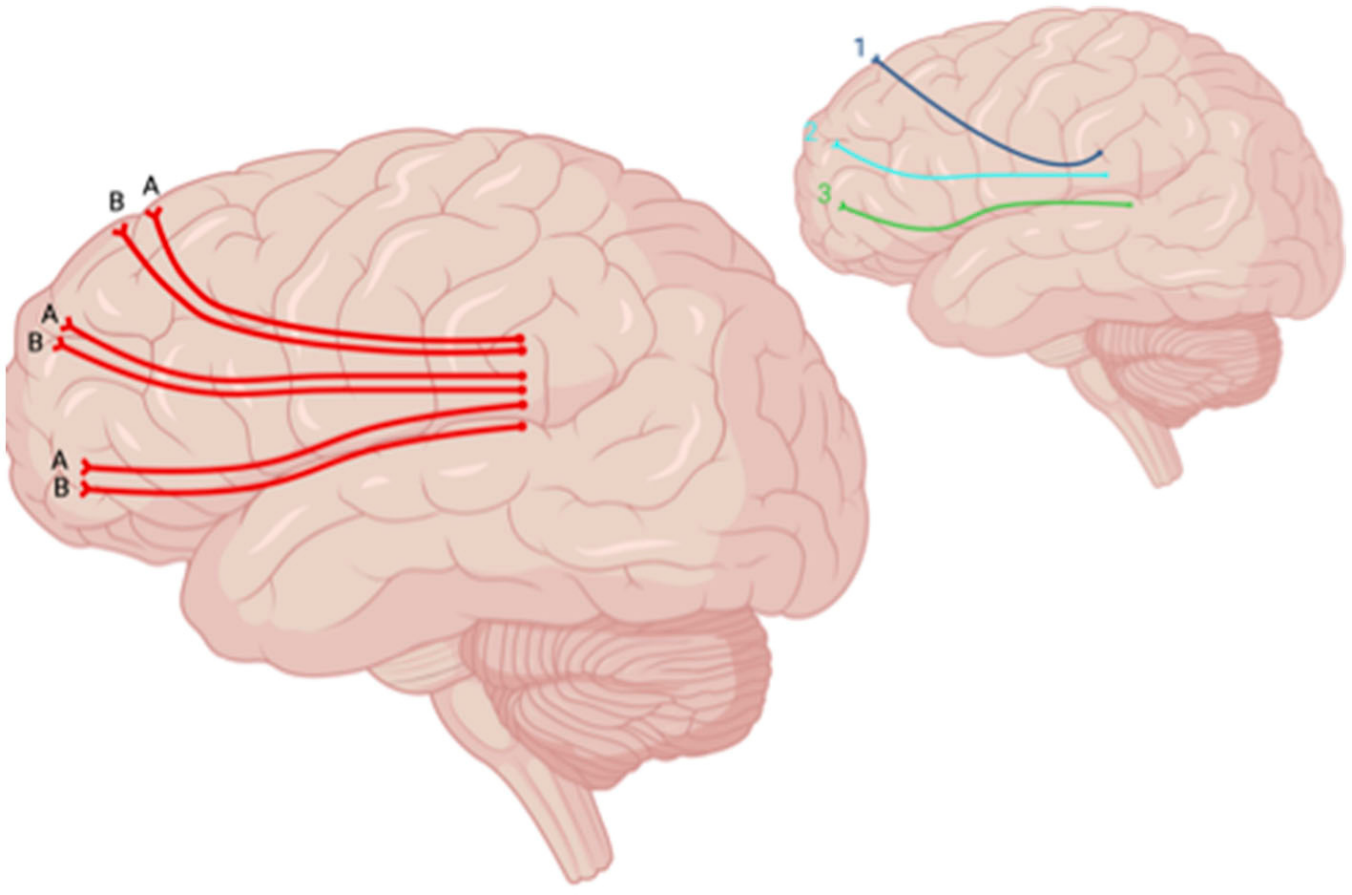
Sagittal lateral view of reported FA results in WM studies. The figure shows reported FA results from the following included studies: (A) Choi and Jang (2021), (B) Jolly et al. (2019). Each number refers to an anatomical WM tract: (1) DLPFTT, (2) VLPFTT, (3) OPFTT. Red lines indicate reported decreases in FA. Created with BioRender.com, RRID:SCR_018361.

The DTI metric of functional anisotropy (FA) was most commonly explored in included studies. Decreased FA was noted in the dorsolateral prefronto-thalamic tract (DLPFTT), ventrolateral prefronto-thalamic tract (VLPFTT), orbital prefronto-thalamic tract (OPFTT), uncinate fasciculus (UF), and cingulum in case studies examining patients with depression post-TBI that were assessed at multiple time points after initial injury (i.e., 3 months, 2 years, and 8 years) (Choi and Jang, 2021; Jang et al., 2016) (Figure 5). Choi and Jang (2021) also reported a decrease in tract volume of the abovementioned WM tracts. A case study by Jang et al. (2016) reported partial tearing of the right DLPFTT and thinning in the left DLPFTT where the individual experienced depression within 2 years post-TBI.

**Figure 6:**
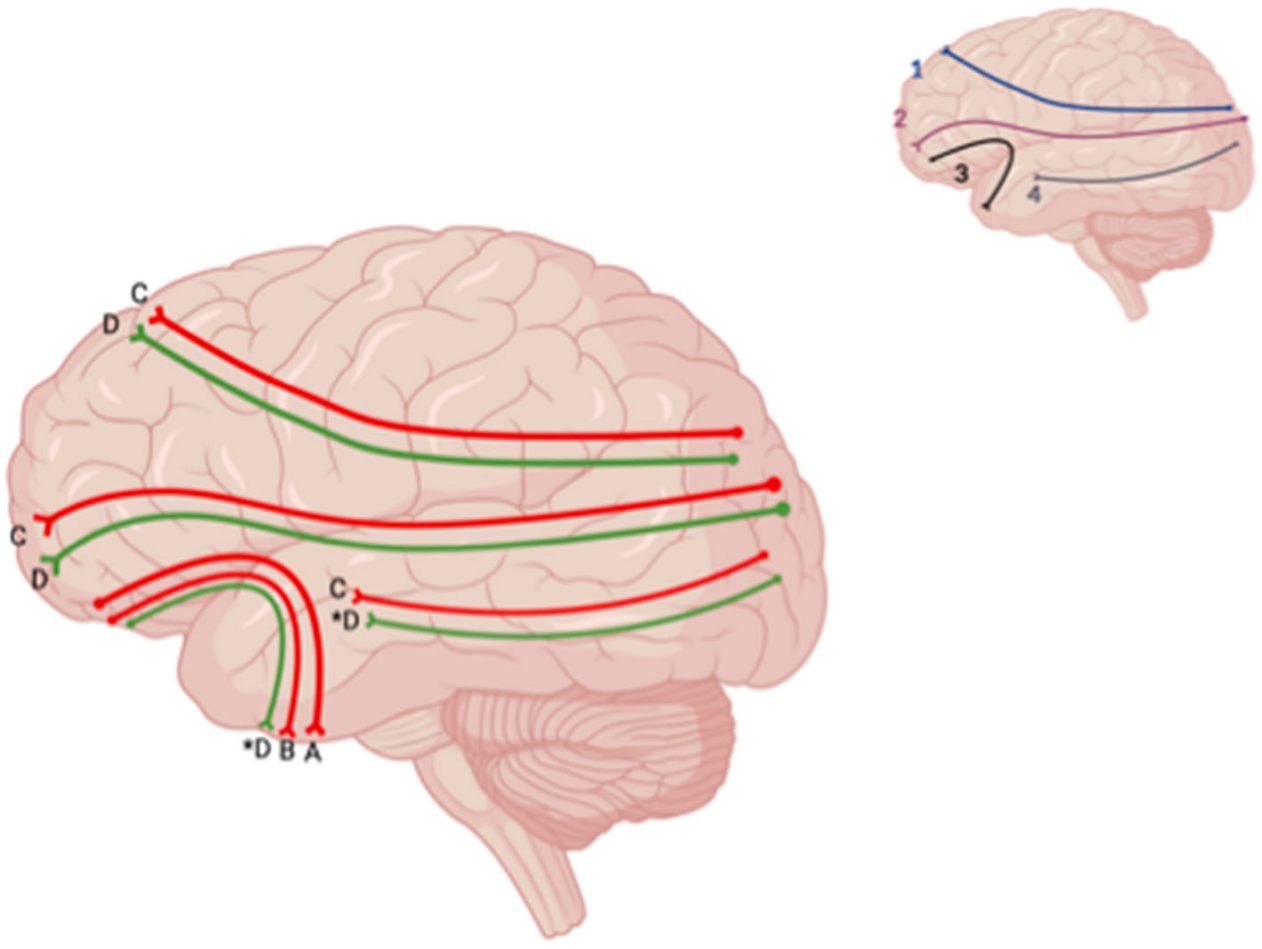
Sagittal lateral view of reported FA results from WM studies. The figure shows reported FA results from the following included studies: (A) Choi and Jang (2021), (B) Jolly et al. (2019), (C) Matthews et al., 2012, (D) Huang et al. (2022). Each number refers to an anatomical WM tract: (1) SLF, (2) IFOF, (3) IFL. Red lines indicate reported decreases in FA. Green lines indicate reported increases in FA. Letters with an asterix (*) indicate a reported relationship with FA in one hemisphere only: (4D*) increase in FA in the right ILF, (3D*) increase in FA in the left UF. Created with BioRender.com, RRID:SCR_018361.

**Figure 7:**
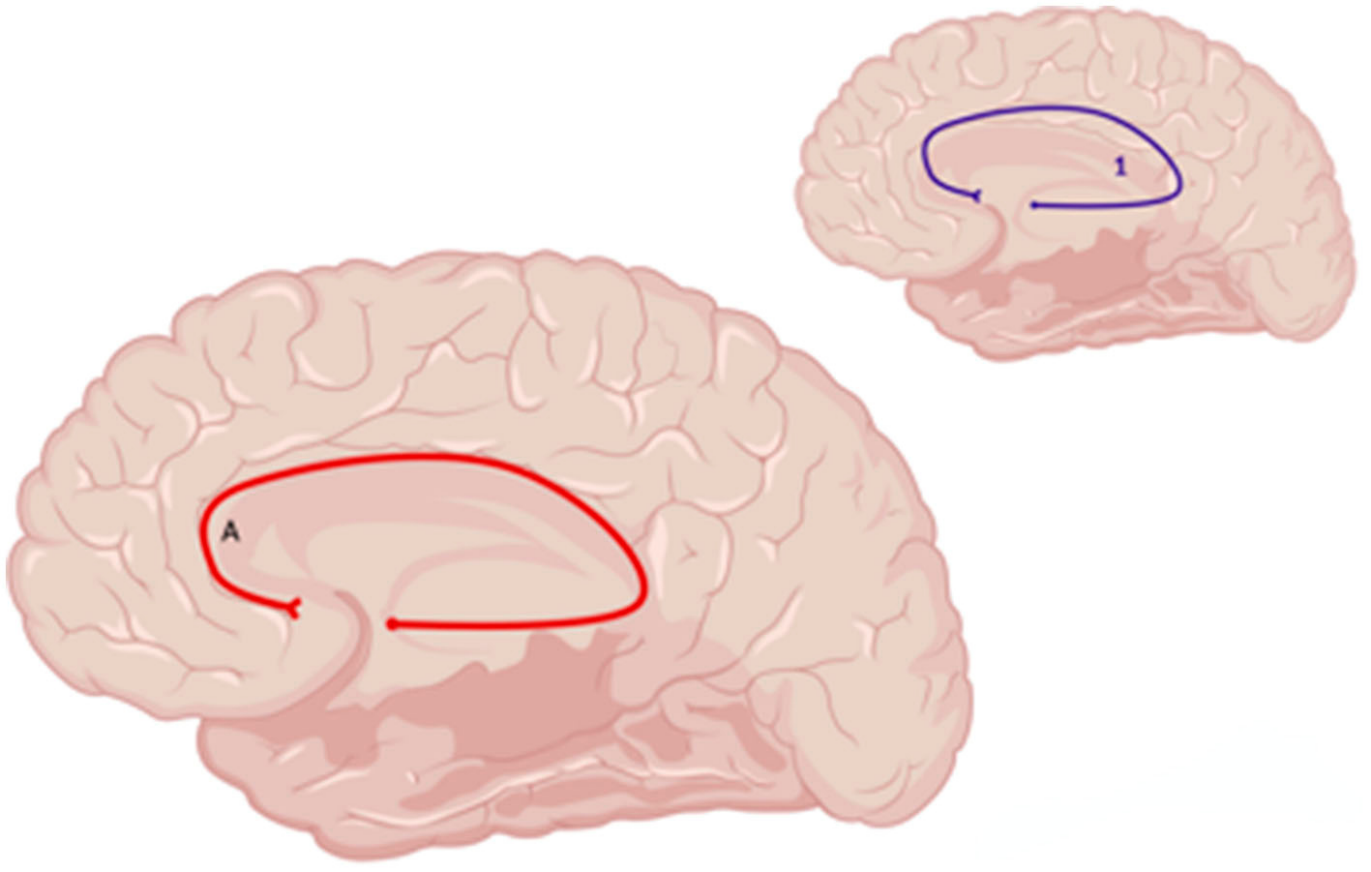
Sagittal medial view of reported FA results in WM studies. The figure shows reported FA results from the following included studies: (A) Choi and Jang (2021). Each number refers to an anatomical WM tract: (1) Cingulum. Red lines indicate reported decreases in FA. Created with BioRender.com, RRID:SCR_018361.

Decreased FA was also reported in the DLPFTT, VLPFTT, OPFTT, and UF in patients who experienced depression post-TBI relative to TBI patients who did not (Jolly et al., 2019) (Figure 6). When comparing patients who had LOC vs. AOC and experienced depression within a year after initial TBI, a decrease in FA was seen in the brainstem, CC, inferior longitudinal fasciculus (ILF), superior longitudinal fasciculus (SLF), inferior fronto-occipital fasciculus (IFOF), anterior limb of the internal capsule, anterior thalamic radiation, and anterior corona radiata in patients with depression post-TBI who experienced LOC (Matthews et al., 2012) (Figure 7). However, one study reported no significant differences in FA at the whole-brain level between MDD-TBI and non-MDD-TBI patients, where time after initial TBI varied between 6 weeks to 10 years (Maller et al., 2014a).

**Figure 8:**
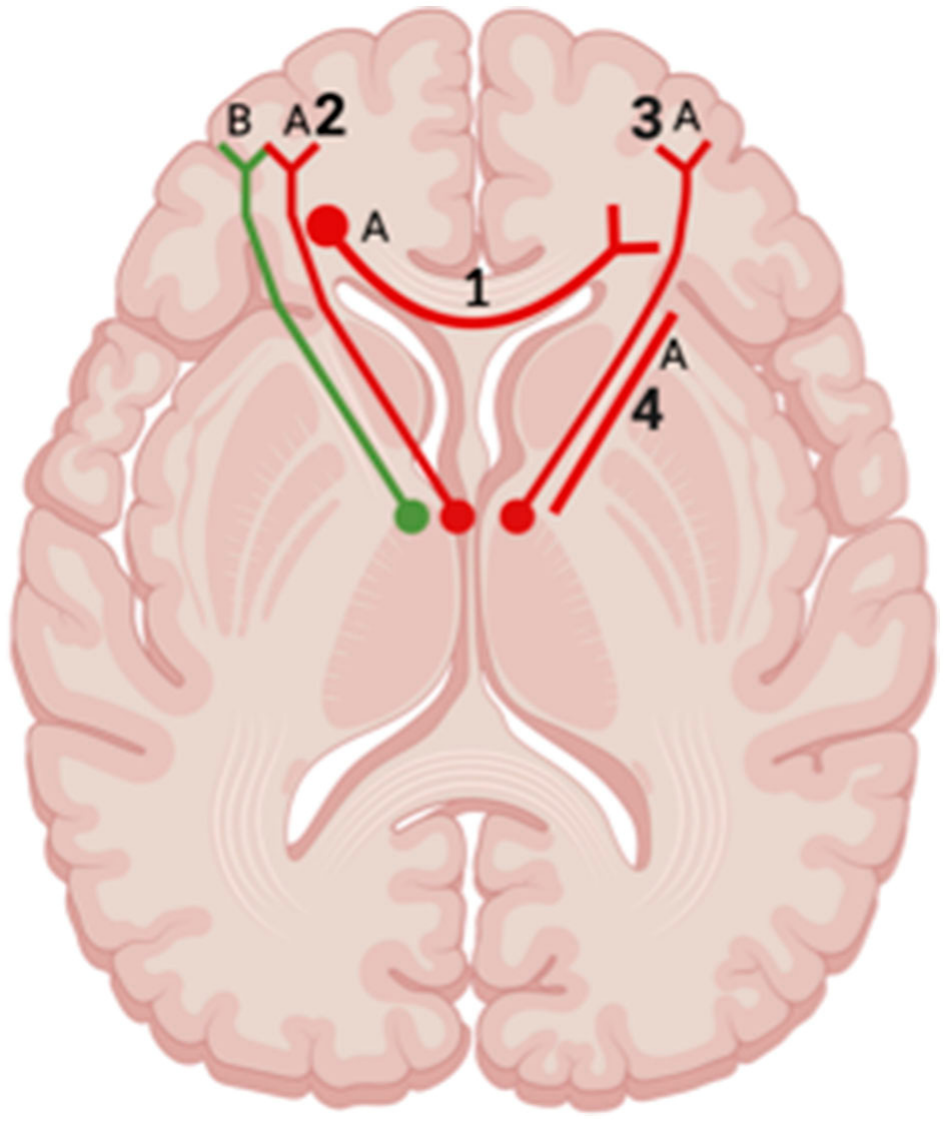
Axial view of reported FA results in WM studies. The figure shows reported FA results from the following included WM studies: **(A)** Matthews et al. (2012), **(B)** Huang et al. (2022). Each number refers to an anatomical WM tract: (1) Corpus Callosum, (2) Left Anterior Thalamic Radiation, (3) Right Anterior Thalamic Radiation, (4) Anterior Limb of Internal Capsule. Red lines indicate reported decreases in FA. Green lines indicate reported increases in FA. Created with BioRender.com, RRID:SCR_018361.

Across studies examining differences between patients with depression post-TBI and HC, one study showed increased FA affecting the bilateral IFOF, SLF, left UF, left anterior thalamic radiation, and right ILF in TBI patients who self-reported depression 7 days after injury compared to HC (Huang et al., 2022) (Figure 8). Jolly et al. (2019) also reported a decrease in FA values within the UF in depression post-TBI patients compared to controls. However, two studies reported no significant differences in FA values at the whole-brain level in TBI groups compared to HC (Maller et al., 2014a; Raikes et al., 2018).

Lastly, there was a distinct difference in reported WM integrity when comparing studies looking at acute (up to 6-months after injury) or chronic phases (6-months after injury) of TBI. For instance, Huang et al. (2022) reported increased FA of the bilateral IFOF, SLF, left UF, left anterior thalamic radiation, and right ILF within 7 days of TBI diagnosis, whereas Matthews et al. (2012) reported decreased FA of the same tracts when looking at depression and TBI one year after diagnosis. Two studies (Choi and Jang, 2021; Raikes et al., 2018) also reported structural WM changes between acute and chronic phases of TBI. In the study by Choi and Jang (2021), the UF had significantly smaller FA values in patients who were assessed 8 years after TBI compared to those assessed 3 months after TBI. Authors linked the decrease in WM integrity to severe depression that patients developed 6 years after TBI. Raikes et al. (2018) examined individuals with mild TBI who were evaluated between 2 weeks to 12 months after their TBI. The results showed no significant differences in any DTI metric in individuals with a history of mild TBI compared to HC.

### 3.6 Gray matter structural differences

Three studies reported structural GM changes associated with depression post-TBI. Spirou et al. (2019) reported no significant volumetric differences in the striatum and dorsomedial prefrontal cortex (DMPFC) while investigating cortico-striatal hyperactivation in individuals with TBI and high vs. low depression scores. Gao et al. (2022) investigated GM changes between TBI and HC groups and reported that bilateral substantia nigra volumes were smaller in post-TBI patients with depression than in HC. In addition, they showed that within 24 h of being diagnosed with TBI, the left substantia nigra was smaller than the right. Lastly, in patients with depression post-TBI compared to HC, Maller et al. (2014b) reported reduced GM volume of the left parietal regions, including the supramarginal and angular gyri and extending into the lateral occipital gyrus. It is also important to acknowledge that two MRI studies used voxel-based morphometry MRI metrics but did not report any GM findings, likely indicative of no significant between-group differences (Jolly et al., 2019; Papadaki et al., 2021).

### 3.7 Functional connectivity differences

Four studies reported alterations in brain FC. McCuddy et al. (2018) reported a significant increase in resting-state FC (rs-FC) between regions of the DMN and the ventral attention network as well as between the DMN and sensorimotor network in TBI patients who were assessed for depression 1 month after injury, compared to HC. This result was also seen in a pairwise comparison between TBI subjects 1 day after injury compared to 1 month after injury (McCuddy et al., 2018). In a study by Gao et al. (2022), decreased rs-FC was seen between the left substantia nigra and bilateral superior frontal gyrus, with signal extending into the right anterior cingulate cortex, in depression post-TBI compared to HC. Decreased rs-FC was also seen between the substantia nigra and left angular gyrus/inferior parietal lobe (Gao et al., 2022).

### 3.8 Hemodynamic differences

Two studies reported changes in BOLD and perfusion outcomes in depression post-TBI. Papadaki et al. (2021) showed decreased resting-state CBF (rs-CBF) in the DLPFC and bilateral hippocampi in TBI patients compared to HC, as well as decreased cerebral blood volume (CBV) in the right DLPFC, right VMPFC, and bilateral hippocampi. Spirou et al. (2019) showed increased activation in the VMPFC and striatum among participants with depression post-TBI receiving a monetary reward during a card-guessing task compared to patients receiving a monetary punishment during a card-guessing game.

### 3.9 Correlation between neuroimaging abnormalities and depression outcomes in TBI

Seven studies investigated the correlation between neuroimaging and depression outcomes. For instance, higher FA and relative anisotropy (RA) values of WM structures, including the left SLF and cingulum, were significantly correlated with the severity of depressive symptoms in mild-TBI patients at the onset of their injury (Matthews et al., 2012). A positive correlation was also seen between Self-Rating Depression Scores (SRDS) and nodal efficiency in the right middle occipital gyrus in mild TBI 7 days post-injury (Huang et al., 2022). One study showed that higher Montgomery-Åsberg Depression Rating Scale (MADRS) scores were associated with a smaller GM volume in the left anterior cingulate, right temporal gyri, and insula at baseline (Maller et al., 2014b). However, a more recent GM study reported opposing results of no significant correlation between GM volume of the middle temporal gyrus, anterior insula, and subgenual anterior cingulate cortex and the severity of symptoms in depression post-TBI patients (Luo et al., 2023).

Hamilton Depression Rating Scale (HAM-D) scores had an inverse association with rs-FC of the DMN with the ventral attention network and fronto-parietal network (McCuddy et al., 2018). One study reported a positive correlation between depression scores and rs-FC between the left anterior insula and bilateral dorsal ACC and rs-FC between the right insula and right medial temporal gyrus (Luo et al., 2023). Luo et al. (2023) also reported a negative correlation between depression scores and rs-FC between the left subgenual anterior cingulate cortex and left medial temporal lobe. In addition, the dorsal posterior cingulate cortex centrality was correlated with the severity of depressive symptoms (Simos et al., 2023).

Lastly, in studies presenting perfusion outcomes, higher depression symptomatology was associated with higher CBF in the putamen bilaterally and increased activation of the VMPFC and left anterior cingulate cortex in depression post-TBI when patients were receiving a monetary reward during a card-guessing game (Spirou et al., 2019). Table 3 summarizes the correlational results reported in the studies.

**Table 3:** Correlational findings for published studies included in the review.

**References**	**Correlation between neuroimaging outcomes and depressive symptoms**
Matthews et al. (2012)	•No significant associations were observed between FA in any of the regions that were different between the LOC and AOC groups and scores on the BDI-II
Maller et al. (2014a)	•N/A
Maller et al. (2014b)	•Among the 14 patients who developed MDD post-TBI, no cluster significantly correlated with MADRS scores after multiple corrections. •However, at p <0.01 before multiple corrections, higher MADRS scores were related to smaller left rostral/caudal anterior cingulate and right temporal gyri and insula •Subcortically, the following regions were statistically correlated with MADRS scores among the 14 patients: left hippocampal volume (r2 = −0.367, p = 0.020), left entorhinal volume (r2 = −0.321, p = 0.043), left thalamus proper (r2 = −0.333, p = 0.036), and right entorhinal volume (r2 = −0.342, p = 0.031).
Jang et al. (2016)	•N/A
McCuddy et al. (2018)	•Inverse association between HAM-D scores and connectivity between regions of the default mode network and regions in the ventral attention network and fronto-parietal network
Raikes et al. (2018)	•No significant correlation within the healthy control group between any of the DTI metrics and any of the self-reported outcomes •In the mild-TBI group, voxels with significant correlations between BDI-FA and BDI-RD metrics were consistently observed bilaterally within the internal capsules, corona radiata, fornix and superior fasiculi
Jolly et al. (2019)	•N/A
Spirou et al. (2019)	•Greater activation in the striatum and vmPFC was associated with higher depression scores when participants were presented with a rewarding outcome
Choi and Jang (2021)	•N/A
Gao et al. (2022)	•In TBI group, FC between the left SN and left angular gyrus was negatively associated with post-traumatic depressive symptoms
Huang et al. (2022)	•Positive correlation between the total SDS scores and nodal efficiency in the right middle occipital gyrus of patients with acute mild-TBI •Negative correlation between the total SDS scores and the shortest nodal path length in the right middle occipital gyrus of patients
Luo et al. (2023)	•There were no significant linear relationships between level of depression and GM volumes of the 26 ROIs
Simos et al. (2023)	•Dorsal PCC centrality correlated with depression symptom severity (CESD score, r = 0.54, p = 0.0006)

AOC, alteration of consciousness; BDI, Beck Depression Inventory; BDI-II, Beck Depression Inventory-II; CESD, Center for Epidemiological Studies Depression Scale; CMDI, Chicago Multiscale Depression Inventory; DSM-5, Diagnostic and Statistical Manual of Mental Disorders, fifth edition; DSM-IV, Diagnostic and Statistical Manual of Mental Disorders, fourth edition; DSM-IV-TR, text revision of Diagnostic and Statistical Manual of Mental Disorders, fourth edition; DTI, diffusion tensor imaging; FA, Functional Anisotropy; FC, functional connectivity; GCS, Glasgow Coma Scale; GM, gray matter; HADS, Hospital Anxiety and Depression Scale; HAM-D, Hamilton Rating Scale for Depression; HAMD-17-Mod, 17-item Hamilton Rating Scale for Depression omitting sleep and weight loss items; HC, healthy control subjects; LOC, loss of consciousness; MADRS, Montgomery-Åsberg Depression Rating Scale; MDD, major depressive disorder; N, sample size; N/A, data not given in the article; PCC, posterior cingulate cortex; RD, radial diffusivity; ROIs, regions of interest; SCID, Structured Clinical Interview for DSM Disorders; SD, standard deviation; SDS, self-reporting depression scale; SN, substantia nigra; SRDS, Zung Self-Rated Depression Scale; TBI, traumatic brain injury; vmPFC, ventromedial prefrontal cortex.

## 4 Discussion

This systematic review provides a synthesis of existing literature on structural and functional MRI studies investigating depression post-TBI. Fourteen studies were included in this review. Studies that investigated TBI and depression had either a between-group HC vs. TBI design or a within-group design (i.e., compared different groups of patients with depression post-TBI). Studies were heterogeneous in terms of the methodology (e.g., comparison groups), assessment tools used to evaluate TBI and depression, and MRI acquisition methods (e.g., scanners, neuroimaging metrics). Thus, a meta-analysis was not feasible due to lack of homogeneity in participants, study designs, and outcomes (Higgins et al., 2019). Overall, included studies indicate several structural and functional changes that could be associated with depression post-TBI. In WM studies, decreases in the integrity of the UF and DLPFTT were highlighted across multiple studies (Choi and Jang, 2021; Jang et al., 2016; Huang et al., 2022; Jolly et al., 2019). In addition, WM studies showed a prominent pattern-like change in WM integrity when comparing acute and chronic TBI. In regard to GM studies, there was no consensus on reported brain regions that are associated with depression post-TBI, as all reported brain regions were supported by no more than one study. Lastly, studies highlighted decreased functional connectivity in the DMN, SN, and sensorimotor network in depression post-TBI patients.

### 4.1 Abnormalities in white matter structure

Despite the fact that most studies included in this review reported WM outcomes, there is scarce evidence for the role of WM in depression post-TBI. Based on the results of the included studies, FA values for WM tracts, including the UF and DLPFTT, had a trend-like increase during the acute phase of TBI (up to 6 months after injury) followed by a decrease in FA in the same WM tracts during the chronic phase of TBI (after 6 months of injury) (Huang et al., 2022; Matthews et al., 2012; Choi and Jang, 2021; Raikes et al., 2018). The UF can be notably highlighted as a tract involved in the development of depression post-TBI, with multiple studies reporting similar findings regarding its impaired integrity in TBI (Mayer et al., 2010; Govindarajan et al., 2016; Veksler et al., 2020). Along with the UF, the DLPFTT was commonly reported to be implicated in depression post-TBI. The UF and DLPFTT are associated with the emotion regulation network (ERN) (Park et al., 2019a; Kaya and McCabe, 2019), which is implicated in the pathophysiology of depression (Park et al., 2019b). Therefore, changes in the integrity of either the UF or DLPFTT seen post-TBI could be implicated in a similar functional pathophysiology that is typical for depressive symptoms. However, among the included studies, there were few correlational analyses outlining the connection between the UF and depression outcomes in TBI. In addition, due to the nature of TBI, physiological phenomena such as secondary injuries (i.e., cytotoxic swelling) should be considered due to their effect on anisotropy (Wilde et al., 2008). Therefore, there is a need for further clarification on the implications of secondary injuries in TBI and whether the phenomena's effect on WM post-TBI is relevant in the development of depression.

Overall, findings reported in this review indicate that there might be a prospective pattern-like change in WM integrity that is time-dependent. Further, the affected WM tracts, such as the UF and DLPFTT, potentially have the capacity to influence the development of depressive symptoms in TBI if these tracts structurally connect key nodes of FC networks that are implicated in depression, such as the ERN.

### 4.2 Abnormalities in gray matter structure, FC, and hemodynamic response

Changes in neural networks, including the ERN, DMN, SN, and sensorimotor network, were also reported in TBI. Similar to depression, changes in key neural networks in TBI were accompanied by structural changes to ROIs for each network. For example, studies not only reported changes in FC of the ERN (McCuddy et al., 2018), but also volumetric and hemodynamic changes in corresponding ROIs, including the substantia nigra, striatum (Gao et al., 2022), DMPFC, VMPFC, and insula (McCuddy et al., 2018; Papadaki et al., 2021). These findings are further supported by studies demonstrating changes in FC between nodes of the DMN and ERN that were correlated with depression scores reflecting mild-to-severe depression symptomatology in TBI patients (McCuddy et al., 2018; Luo et al., 2023; Simos et al., 2023). Therefore, the influence of behaviors associated with dysfunction in the ERN, such as rumination and emotional suppression, may influence the pathogenesis of depression in TBI the same way it is hypothesized to in depression (Compare et al., 2014). Likewise, the function of the SN lies in its ability to filter which stimuli become conscious to the brain (Manoliu et al., 2014). In TBI, dysregulation of this network manifests itself as increased severity of self-reported depressive symptoms in patients (van der Horn et al., 2016). Lastly, studies suggest that sensory processing plays a role in modulating mood states (Canbeyli, 2013), including agitation and feelings of fatigue in depression (Ray et al., 2021). Therefore, reported changes in FC of the sensorimotor network in TBI patients (McCuddy et al., 2018) can suggest impairments in the ability to modulate agitated moods, which leads to increased susceptibility to depressive symptoms among TBI patients.

Overall, the results indicate that depression in TBI may follow the “hypersensitivity” hypothesis where FC, BOLD, and volumetric changes are considered compensatory in the brain's attempt to collect information necessary to return itself back to its normal status (Iraji et al., 2015). This logic agrees with included studies reporting changes in GM volume during the acute phase of TBI. Therefore, FC and structural GM changes may be predictive of depression outcomes in TBI. However, additional research needs to be conducted to validate the reported changes in GM, FC, and hemodynamic outcomes in TBI and strengthen conclusions regarding the correlational results that suggest their association with depression post-TBI.

### 4.3 Similarities and differences in MDD and depression post-TBI

Included studies indicate more potential structural and functional differences between MDD and MDD-TBI. In studies investigating WM in depression post-TBI, there were reports of changes in the integrity of the CC and internal capsule, as also characterized in MDD without TBI (Matthews et al., 2012; van Velzen et al., 2020). However, these structural changes are not reported consistently. Therefore, other WM structural changes that appear more consistently reported in the included studies, as seen with the UF and DLPTT, could serve as neural markers to distinguish depression post-TBI and MDD without TBI. GM studies included in our review did not indicate any strong associations between volumetric GM changes and depression post-TBI in any particular brain region. In addition, our review did not note any volumetric changes in brain regions that typically display altered GM volume in MDD without TBI, such as the amygdala and parahippocampal gyrus (Romeo et al., 2024). Therefore, more research is needed to characterize which changes in GM are linked to depression post-TBI to determine if they are, in fact, distinct from MDD. MDD and depression post-TBI are both associated with functional changes to the DMN. For example, both demonstrate elevated FC between the DMN and SN (McCuddy et al., 2018; Dai et al., 2019). However, depression post-TBI slightly differs from MDD without TBI as it also displays elevated FC between the DMN and sensorimotor network (McCuddy et al., 2018), which might be a notable feature of TBI. However, these functional changes are not consistently reported across studies and only serve as a foundation for future investigation with larger TBI cohorts. Therefore, more research is needed to verify these reported associations before they can be considered as a valid distinction between depression post-TBI and MDD without TBI.

### 4.4 Strengths and limitations

A strength of this review lies in its inclusion of studies that permitted comorbid anxiety with TBI (Gao et al., 2022; Papadaki et al., 2021). This improves the generalizability of our synthesis, as anxiety is highly comorbid with depression, as well as with TBI (Al-Kader et al., 2022). In addition, this review succinctly summarizes both functional and structural neuroimaging and correlational outcomes to present a comprehensive update of the current literature on depression post-TBI and encourage the field's transition to utilizing MRI techniques in study designs.

There are several limitations to the current review. The small number of studies included affects the reliability of presented results. For example, there were a few occasions where conclusions about a specific set of results were made based on a limited number of studies that reported on that specific outcome (i.e., GM, WM, and FC outcomes). In addition, some studies had a very small number of participants. Therefore, the results reported in this review should be considered as a starting point for investigating the brain-based correlates of depression and TBI until more evidence becomes available. In addition, within the pool of included studies, there was a vast amount of heterogeneity in the study populations and methodologies used. There was variability in whether studies enrolled TBI patients from populations with specified TBIs, such as concussions, or individuals who were grouped into TBI categories (i.e., mild, moderate, severe) based on TBI scales and classification systems (i.e., GCS and Mayo Clinic classification system). In addition, most studies did not categorize patients based on the severity of TBI (e.g., mild, moderate, severe) but rather defined TBI as any individual with a GCS of <13. This specification is important as patients with mild TBI (e.g., a concussion) experience different structural changes to the brain compared to a patient with severe TBI (i.e., if a patient is unconscious for more than 6 h) (Mckee and Daneshvar, 2015). This is significant, as the development of depression in TBI patients may also differ. Therefore, studies reporting no significant findings could have been influenced by the lack of specificity when categorizing TBI in patients. Variations in TBI diagnosis limit the reliability of presented results since some studies did not include the GCS, which is a required component for studies on head injury based on the National Institutes of Health policies (Jain and Iverson, 2023). Studies were also variable in terms of the group comparisons they conducted, with analyses comparing TBI patients with HC or subsets within the TBI population (LOC, AOC, high or low depression symptomatology). There was also variability in how studies diagnosed depression. Many studies used validated depression scales. However, they did not verify these diagnoses with structural clinical reviews (SCR), which is a standard in psychiatric clinical practice (Sanchez-Villegas et al., 2008). Overall, the lack of standardized approaches to studying depression in TBI impacts how the presented results can be interpreted. Therefore, to improve the interpretation of reported results in depression post-TBI studies, it is crucial to start integrating the use of SCR to validate depression diagnoses in TBI patients.

### 4.5 Future directions and conclusion

The current systematic review provides insight into the neural underpinnings of depression post-TBI. Generally, evidence supporting changes in structural WM and GM, FC, and hemodynamic outcomes is limited. Therefore, additional studies should be conducted to gain a more comprehensive understanding of the alterations that occur in the brain during TBI and how they are related to the presentation of depression in participants. However, this systematic review provides an initial insight into potential neuroimaging-based correlates of depression post-TBI. An investigation into the structural and functional changes in depression with TBI would contribute to a better understanding of how diffuse axonal injury (DAI) is implicated in the development of psychiatric disorders in TBI (Aldossary et al., 2019). DAI is associated with several neurocognitive outcomes post-TBI. Research suggests that observed lesion localization and volume are predictive of the degree of poor neurocognitive outcomes (Rabinowitz and Smith, 2016). Following this trend, the same prognostic capacity could also be applied in predicting psychiatric outcomes. The current review supports this goal as it indicates that most studies investigating depression in TBI report changes in structural WM. Accompanying this approach, more studies are needed to delineate the characteristic structural and functional changes that occur in both acute and chronic TBI. In addition, by characterizing physiological changes, further distinctions can be made on which structural or functional changes are associated with other phenomena that occur in TBI, including neuroinflammation. To accomplish this objective, barriers associated with studying FC in post-TBI depression need to be uncovered to allow for informative neuroimaging outcomes to be reported in future studies (Medaglia, 2017). In addition, the ranging severity of TBI (mild, moderate, or severe) studied in post-TBI depression research raises the question of how the etiology of depression can vary in individuals who also vary in TBI severity. If such a relationship exists, it suggests that studies with certain groups of TBI patients may yield different significant findings with regard to the underlying mechanisms of depression in different TBI severity groups. Therefore, addressing these issues would be the next step toward improving protocols used in post-TBI depression research in the interest of advancing the field.

Overall, evidence from 14 published studies indicates altered structural and functional activity in the brains of individuals with depression post-TBI. This is the first review to systematically examine existing MRI studies on depression outcomes in TBI. Here, we have identified current trends in structural WM and GM, FC, and BOLD in the context of the presentation of depression in TBI patients. In summary, current research in the field indicates evidence supporting that the networks central to depression, including the DMN and ERN, are also affected in depression post-TBI. WM tracts that are associated with the structural connectivity of these networks, such as the UF and DLPFTT, may also play a role in the development of depression post-TBI.

## Data Availability

The original contributions presented in the study are included in the article/Supplementary material, further inquiries can be directed to the corresponding author.
