# Supplementary material for: Brain-based correlates of depression and traumatic brain injury: a systematic review of structural and functional magnetic resonance imaging studies
Source: Front Neuroimaging. 2024 Nov 5;3:1465612. doi: 10.3389/fnimg.2024.1465612 (PMC11573519; doi:10.3389/fnimg.2024.1465612)
Supplement: Supplementary file 1 [file Data_Sheet_1.DOCX]

**Appendix A**

Ovid Activity Search Strategy

Embase Classic+Embase <1947 to 2022 November 15>

APA PsycInfo <1806 to November Week 1 2022>

Ovid MEDLINE(R) ALL <1946 to November 15, 2022>

1 (brain injuries, traumatic or brain injury, traumatic or brain trauma or brain traumas or encephalopathies, traumatic or encephalopathy, traumatic or injury, brain, traumatic or "tbi (traumatic brain injuries)" or "tbi (traumatic brain injury)" or "tbis (traumatic brain injuries)" or trauma, brain or traumas, brain or traumatic brain injuries or traumatic brain injury or traumatic encephalopathies or traumatic encephalopathy).mp. 158138

2 exp Craniocerebral Trauma/ or Craniocerebral Trauma.mp. or (exp Brain Injuries, Traumatic/ or Brain Injuries, Traumatic.mp.) or (exp Brain Injury, Chronic/ or Brain Injury, Chronic.mp.) or (exp Epilepsy, Post-Traumatic/ or Epilepsy, Post-Traumatic.mp.) or (exp Pneumocephalus/ or Pneumocephalus.mp.) or (exp Brain Concussion/ or Brain Concussion.mp.) or (exp Intracranial Hemorrhage, Traumatic/ or Intracranial Hemorrhage, Traumatic.mp.) or (exp Skull Fractures/ or Skull Fractures.mp.) 700607

3 exp Brain Contusion/ 4572

4 exp Brain Stem Hemorrhage, Traumatic/ or exp Cerebral Hemorrhage, Traumatic/ or exp Diffuse Axonal Injury/ or exp Cerebrospinal Fluid Otorrhea/ or exp Cerebrospinal Fluid Rhinorrhea/ or exp Head Injuries, Penetrating/ or exp Hematoma, Epidural, Cranial/ or exp Hematoma, Subdural/ or exp Subarachnoid Hemorrhage, Traumatic/ or exp Skull Fracture, Depressed/ 551159

5 exp Intracranial Hemorrhage, Traumatic/ 187880

6 1 or 2 or 3 or 4 or 5 759821

7 exp Depressive Disorder, Major/ or Depressive Disorder, Major.mp. or exp Unipolar Depress*/ or (exp Depressive Disorder, Treatment-Resistant/ or Depressive Disorder, Treatment-Resistant.mp.) or Severe Depress*.mp. or Moderate Depress*.mp. [mp=ti, ab, hw, tn, ot, dm, mf, dv, kf, fx, dq, tc, id, tm, bt, nm, ox, px, rx, ui, sy] 165227

8 ((major adj1 depress*) or MDD).ti,ab,kf,kw. 185014

9 (depressive disorder* or depressive symptom* or depression symptom*).ti,ab,kf,kw. 353615

10 (depress* not (postpartum or seasonal or manic or hypomanic or puerperal or organic or dysthymi* or chronic or bipolar or substance?induced or drug?induced or substance?related or drug?related or psychotic or chemical or synaptic or inbreeding or cortical spreading or economic or involutional or postpartum or post-natal or tooth or winter or homosynaptic or spreading or ventilatory or respiratory)).kf,kw. 203981

11 ((Severe adj1 depress*) or TRD).ti,ab,kf,kw. 31599

12 7 or 8 or 9 or 11 481705

13 10 and 12 102533

14 (brain imaging, functional or brain imagings, functional or functional brain imaging or functional brain imagings or functional neuroimaging or imaging, functional brain or imagings, functional brain or neuroimaging, functional).mp. 42179

15 (functional mr or functional mri or functional mris or functional magnetic resonance imaging or mri, functional or mris, functional or magnetic resonance imaging, functional or fmri).mp. 243115

16 (fMR or fMRI or rsfMRI).mp. [mp=ti, ab, hw, tn, ot, dm, mf, dv, kf, fx, dq, tc, id, tm, bt, nm, ox, px, rx, ui, sy] 173674

17 (functional magnetic resonance imag* or functional magnetic resonance scan or functional magnetic resonance neuroimag* or functional MRI or functional MR).mp. 203355

18 (functional cerebral localization or functional cerebral localizations).mp. 4

19 (BOLD or BOLD-contrast or BOLD-contrast imag*).mp. [mp=ti, ab, hw, tn, ot, dm, mf, dv, kf, fx, dq, tc, id, tm, bt, nm, ox, px, rx, ui, sy] 47622

20 (Blood oxygen level dependent or Blood-oxygen-level-dependent).ti,ab,kf,kw. 12471

21 functional magnetic resonance.mp. 183092

22 (functional and (MR or MRI or magnetic resonance) and (scan* or imag* or neuroimag*)).mp. 342448

23 (hemodynamic response or haemodynamic response).mp. 16419

24 exp *Echo-Planar Imaging/ or Echo-Planar Imaging.mp. 15885

25 exp *Functional Neuroimaging/ or Functional Neuroimaging.mp. 73437

26 14 or 15 or 16 or 17 or 18 or 19 or 20 or 21 or 22 or 23 or 24 or 25 446184

27 6 and 13 and 26 47

28 limit 27 to english language 46

29 limit 28 to human 44

30 remove duplicates from 29 40

Ovid Functional Connectivity Search Strategy

Embase Classic+Embase <1947 to 2022 November 15>

APA PsycInfo <1806 to November Week 1 2022>

Ovid MEDLINE(R) ALL <1946 to November 15, 2022>

1 (brain injuries, traumatic or brain injury, traumatic or brain trauma or brain traumas or encephalopathies, traumatic or encephalopathy, traumatic or injury, brain, traumatic or "tbi (traumatic brain injuries)" or "tbi (traumatic brain injury)" or "tbis (traumatic brain injuries)" or trauma, brain or traumas, brain or traumatic brain injuries or traumatic brain injury or traumatic encephalopathies or traumatic encephalopathy).mp. 158138

2 exp Craniocerebral Trauma/ or Craniocerebral Trauma.mp. or (exp Brain Injuries, Traumatic/ or Brain Injuries, Traumatic.mp.) or (exp Brain Injury, Chronic/ or Brain Injury, Chronic.mp.) or (exp Epilepsy, Post-Traumatic/ or Epilepsy, Post-Traumatic.mp.) or (exp Pneumocephalus/ or Pneumocephalus.mp.) or (exp Brain Concussion/ or Brain Concussion.mp.) or (exp Intracranial Hemorrhage, Traumatic/ or Intracranial Hemorrhage, Traumatic.mp.) or (exp Skull Fractures/ or Skull Fractures.mp.) 700607

3 exp Brain Contusion/ 4572

4 exp Brain Stem Hemorrhage, Traumatic/ or exp Cerebral Hemorrhage, Traumatic/ or exp Diffuse Axonal Injury/ or exp Cerebrospinal Fluid Otorrhea/ or exp Cerebrospinal Fluid Rhinorrhea/ or exp Head Injuries, Penetrating/ or exp Hematoma, Epidural, Cranial/ or exp Hematoma, Subdural/ or exp Subarachnoid Hemorrhage, Traumatic/ or exp Skull Fracture, Depressed/ 551159

5 exp Intracranial Hemorrhage, Traumatic/ 187880

6 1 or 2 or 3 or 4 or 5 759821

7 exp Depressive Disorder, Major/ or Depressive Disorder, Major.mp. or exp Unipolar Depress*/ or (exp Depressive Disorder, Treatment-Resistant/ or Depressive Disorder, Treatment-Resistant.mp.) or Severe Depress*.mp. or Moderate Depress*.mp. [mp=ti, ab, hw, tn, ot, dm, mf, dv, kf, fx, dq, tc, id, tm, bt, nm, ox, px, rx, ui, sy] 165227

8 ((major adj1 depress*) or MDD).ti,ab,kf,kw. 185014

9 (depressive disorder* or depressive symptom* or depression symptom*).ti,ab,kf,kw. 353615

10 (depress* not (postpartum or seasonal or manic or hypomanic or puerperal or organic or dysthymi* or chronic or bipolar or substance?induced or drug?induced or substance?related or drug?related or psychotic or chemical or synaptic or inbreeding or cortical spreading or economic or involutional or postpartum or post-natal or tooth or winter or homosynaptic or spreading or ventilatory or respiratory)).kf,kw. 203981

11 ((Severe adj1 depress*) or TRD).ti,ab,kf,kw. 31599

12 7 or 8 or 9 or 11 481705

13 10 and 12 102533

14 Magnetic Resonance Imaging/ or MRI.mp. or Functional Magnetic Resonance Imaging.mp. or fMRI.mp. [mp=ti, ab, hw, tn, ot, dm, mf, dv, kf, fx, dq, tc, id, tm, bt, nm, ox, px, rx, ui, sy] 1626286

15 exp *Functional Neuroimaging/ or Functional Neuroimaging.mp. 73437

16 (FC or functional connectivity or functional connectome or effective connectivity or effective connectome).mp. 223745

17 (dynamic functional connectivity or DFC).mp. 3650

18 14 or 15 or 16 or 17 1816596

19 6 and 13 and 18 134

20 limit 19 to english language 131

21 limit 20 to human 125

22 remove duplicates from 21 114

Ovid White Matter Search Strategy

Embase Classic+Embase <1947 to 2022 November 15>

APA PsycInfo <1806 to November Week 1 2022>

Ovid MEDLINE(R) ALL <1946 to November 15, 2022>

1 (brain injuries, traumatic or brain injury, traumatic or brain trauma or brain traumas or encephalopathies, traumatic or encephalopathy, traumatic or injury, brain, traumatic or "tbi (traumatic brain injuries)" or "tbi (traumatic brain injury)" or "tbis (traumatic brain injuries)" or trauma, brain or traumas, brain or traumatic brain injuries or traumatic brain injury or traumatic encephalopathies or traumatic encephalopathy).mp. 158138

2 exp Craniocerebral Trauma/ or Craniocerebral Trauma.mp. or (exp Brain Injuries, Traumatic/ or Brain Injuries, Traumatic.mp.) or (exp Brain Injury, Chronic/ or Brain Injury, Chronic.mp.) or (exp Epilepsy, Post-Traumatic/ or Epilepsy, Post-Traumatic.mp.) or (exp Pneumocephalus/ or Pneumocephalus.mp.) or (exp Brain Concussion/ or Brain Concussion.mp.) or (exp Intracranial Hemorrhage, Traumatic/ or Intracranial Hemorrhage, Traumatic.mp.) or (exp Skull Fractures/ or Skull Fractures.mp.) 700607

3 exp Brain Contusion/ 4572

4 exp Brain Stem Hemorrhage, Traumatic/ or exp Cerebral Hemorrhage, Traumatic/ or exp Diffuse Axonal Injury/ or exp Cerebrospinal Fluid Otorrhea/ or exp Cerebrospinal Fluid Rhinorrhea/ or exp Head Injuries, Penetrating/ or exp Hematoma, Epidural, Cranial/ or exp Hematoma, Subdural/ or exp Subarachnoid Hemorrhage, Traumatic/ or exp Skull Fracture, Depressed/ 551159

5 exp Intracranial Hemorrhage, Traumatic/ 187880

6 1 or 2 or 3 or 4 or 5 759821

7 exp Depressive Disorder, Major/ or Depressive Disorder, Major.mp. or exp Unipolar Depress*/ or (exp Depressive Disorder, Treatment-Resistant/ or Depressive Disorder, Treatment-Resistant.mp.) or Severe Depress*.mp. or Moderate Depress*.mp. [mp=ti, ab, hw, tn, ot, dm, mf, dv, kf, fx, dq, tc, id, tm, bt, nm, ox, px, rx, ui, sy] 165227

8 ((major adj1 depress*) or MDD).ti,ab,kf,kw. 185014

9 (depressive disorder* or depressive symptom* or depression symptom*).ti,ab,kf,kw. 353615

10 (depress* not (postpartum or seasonal or manic or hypomanic or puerperal or organic or dysthymi* or chronic or bipolar or substance?induced or drug?induced or substance?related or drug?related or psychotic or chemical or synaptic or inbreeding or cortical spreading or economic or involutional or postpartum or post-natal or tooth or winter or homosynaptic or spreading or ventilatory or respiratory)).kf,kw. 203981

11 ((Severe adj1 depress*) or TRD).ti,ab,kf,kw. 31599

12 7 or 8 or 9 or 11 481705

13 10 and 12 102533

14 Magnetic Resonance Imaging/ 1067898

15 (dti mri or diffusion tensor imaging or diffusion tensor mri or diffusion tensor mris or diffusion tensor magnetic resonance imaging or diffusion tractography or imaging, diffusion tensor or mri, diffusion tensor or tractography, diffusion).mp. 62670

16 exp andd/ or Diffusion Magnetic Resonance Imaging.mp. or (exp Diffusion Tensor Imaging/ or Diffusion Tensor Imaging.mp.) 132890

17 exp *Connectome/ or Connectome.mp. 25818

18 exp *Neural Pathways/ or Neural Pathways.mp. or (exp *Afferent Pathways/ or Afferent Pathways.mp.) or (exp *Efferent Pathways/ or Efferent Pathways.mp.) 224717

19 exp *White Matter/ or White Matter.mp. 202971

20 (interconnection, neural or interconnections, neural or neural interconnection or neural interconnections or neural pathway or neural pathways or pathway, neural or pathways, neural).mp. 87907

21 (anatomical connection* or white matter connection* or anatomical interconnection* or white matter interconnection* or anatomical tract* or white matter tract* or anatomical projection* or white matter projection* or anatomical commissure* or white matter commissure* or anatomical pathway* or white matter pathway*).mp. [mp=ti, ab, hw, tn, ot, dm, mf, dv, kf, fx, dq, tc, id, tm, bt, nm, ox, px, rx, ui, sy] 20842

22 tractography.mp. 20985

23 (cerebellar white matter or cerebellar white matters or matter, cerebellar white or matter, white or matters, cerebellar white or matters, white or white matter or white matter, cerebellar or white matters or white matters, cerebellar).mp. 202518

24 (anatomical connectivity or white matter connectivity).mp. 4085

25 (DTI or DWI).mp. 71415

26 (dwi mri or diffusion-weighted imaging or diffusion-weighted mri or diffusion-weighted mris or diffusion-weighted magnetic resonance imaging or imaging, diffusion-weighted or mri, diffusion-weighted).mp. 78744

27 exp *Brain Mapping/ or Brain Mapping.mp. 182498

28 (brain connectomic or brain connectomics or connectome or connectome, human or connectome mapping or connectome mappings or connectome project, human or connectome projects, human or connectomes or connectomes, human or connectomic or connectomic, brain or connectomics or connectomics, brain or human connectome or human connectome project or human connectome projects or human connectomes or mapping, connectome or mappings, connectome).mp. 27299

29 14 or 15 or 16 or 17 or 18 or 19 or 20 or 21 or 22 or 23 or 24 or 25 or 26 or 27 or 28 1612334

30 6 and 13 and 29 120

31 limit 30 to english language 116

32 limit 31 to human 112

33 remove duplicates from 32 107

Ovid Grey Matter Search Strategy

Embase Classic+Embase <1947 to 2022 November 15>

APA PsycInfo <1806 to November Week 1 2022>

Ovid MEDLINE(R) ALL <1946 to November 15, 2022>

1 (brain injuries, traumatic or brain injury, traumatic or brain trauma or brain traumas or encephalopathies, traumatic or encephalopathy, traumatic or injury, brain, traumatic or "tbi (traumatic brain injuries)" or "tbi (traumatic brain injury)" or "tbis (traumatic brain injuries)" or trauma, brain or traumas, brain or traumatic brain injuries or traumatic brain injury or traumatic encephalopathies or traumatic encephalopathy).mp. 158138

2 exp Craniocerebral Trauma/ or Craniocerebral Trauma.mp. or (exp Brain Injuries, Traumatic/ or Brain Injuries, Traumatic.mp.) or (exp Brain Injury, Chronic/ or Brain Injury, Chronic.mp.) or (exp Epilepsy, Post-Traumatic/ or Epilepsy, Post-Traumatic.mp.) or (exp Pneumocephalus/ or Pneumocephalus.mp.) or (exp Brain Concussion/ or Brain Concussion.mp.) or (exp Intracranial Hemorrhage, Traumatic/ or Intracranial Hemorrhage, Traumatic.mp.) or (exp Skull Fractures/ or Skull Fractures.mp.) 700607

3 exp Brain Contusion/ 4572

4 exp Brain Stem Hemorrhage, Traumatic/ or exp Cerebral Hemorrhage, Traumatic/ or exp Diffuse Axonal Injury/ or exp Cerebrospinal Fluid Otorrhea/ or exp Cerebrospinal Fluid Rhinorrhea/ or exp Head Injuries, Penetrating/ or exp Hematoma, Epidural, Cranial/ or exp Hematoma, Subdural/ or exp Subarachnoid Hemorrhage, Traumatic/ or exp Skull Fracture, Depressed/ 551159

5 exp Intracranial Hemorrhage, Traumatic/ 187880

6 1 or 2 or 3 or 4 or 5 759821

7 exp Depressive Disorder, Major/ or Depressive Disorder, Major.mp. or exp Unipolar Depress*/ or (exp Depressive Disorder, Treatment-Resistant/ or Depressive Disorder, Treatment-Resistant.mp.) or Severe Depress*.mp. or Moderate Depress*.mp. [mp=ti, ab, hw, tn, ot, dm, mf, dv, kf, fx, dq, tc, id, tm, bt, nm, ox, px, rx, ui, sy] 165227

8 ((major adj1 depress*) or MDD).ti,ab,kf,kw. 185014

9 (depressive disorder* or depressive symptom* or depression symptom*).ti,ab,kf,kw. 353615

10 (depress* not (postpartum or seasonal or manic or hypomanic or puerperal or organic or dysthymi* or chronic or bipolar or substance?induced or drug?induced or substance?related or drug?related or psychotic or chemical or synaptic or inbreeding or cortical spreading or economic or involutional or postpartum or post-natal or tooth or winter or homosynaptic or spreading or ventilatory or respiratory)).kf,kw. 203981

11 ((Severe adj1 depress*) or TRD).ti,ab,kf,kw. 31599

12 7 or 8 or 9 or 11 481705

13 10 and 12 102533

14 Magnetic Resonance Imaging/ 1067898

15 (T1 or T2 or T2* or T1-weighted or T2-weighted or T2*-weighted or T1 weighted or T2 weighted or T2* weighted).mp. 628373

16 (structural brain imag* or structural neuroimag*).mp. 5219

17 (anatomical MRI or anatomical MR or anatomical magnetic resonance imag* or anatomical magnetic resonance scan or anatomical magnetic resonance neuroimag*).mp. 2561

18 (FLAIR or fluid attenuated inversion recovery).mp. 26794

19 (magnetisation transfer imag* or magnetization transfer imag* or MTI).mp. 3672

20 (proton density imag* or proton density neuroimag* or proton density scan* or proton density brain imag*).mp. 382

21 (structural MRI or structural MR or structural magnetic resonance imag* or structural magnetic resonance scan* or structural magnetic resonance neuroimag*).mp. 23612

22 (gray matter or gray matters or grey matter or grey matters or matter, gray or matter, grey or matters, gray or matters, grey).mp. 112453

23 exp *Gray Matter/ or Gray Matter.mp. 100437

24 (structural MRI or structural MR or structural magnetic resonance imag* or structural magnetic resonance scan* or structural magnetic resonance neuroimag*).mp. 23612

25 (morpholog* or morphometr*).mp. 1827015

26 14 or 15 or 16 or 17 or 18 or 19 or 20 or 21 or 22 or 23 or 24 or 25 3409929

27 6 and 13 and 26 103

28 limit 27 to english language 100

29 limit 28 to human 98

30 remove duplicates from 29 93
