# Supplementary material for: Brain-based correlates of depression and traumatic brain injury: a systematic review of structural and functional magnetic resonance imaging studies
Source: Front Neuroimaging. 2024 Nov 5;3:1465612. doi: 10.3389/fnimg.2024.1465612 (PMC11573519; doi:10.3389/fnimg.2024.1465612)
Supplement: Supplementary file 2 [file Data_Sheet_2.DOCX]

**Appendix B**

**Table S1. JBI Checklist for Assessment of Article Quality**

**Colour Coding:     Yes      No            Unclear             N/A**

| **Case Control Studies** | | | | | | | | | |
| --- | --- | --- | --- | --- | --- | --- | --- | --- | --- |
| **Study** | **Q1** | **Q2** | **Q3** | **Q4** | **Q6** | **Q7** | **Q8** | **Q9** | **Q10** |
| McCuddy et al., 2018 | Yes | Yes | Yes | Yes | Yes | Yes | Yes | Yes | Unclear |
| Papidaki et al., 2021 | No | Yes | Yes | Yes | Yes | No | Yes | Yes | Yes |
| Spirou et al., 2019 | Yes | Yee | Yes | Yes | Yes | Yes | Yes | Yes | Yes |
| Choi et al., 2021 | N/A | N/A | N/A | Yes | Yes | Yes | Yes | Yes | Yes |
| Raikes et al., 2018 | Yes | Yes | Yes | Yes | Yes | Yes | Yes | Yes | Yes |
| Mathews et al., 2012 | Yes | Yes | Yes | Yes | Yes | Yes | Yes | Yes | Yes |
| Jolly et al., 2019 | Yes | Yes | Yes | Yes | Yes | Yes | Yes | Yes | Yes |
| Huang et al., 2022 | Yes | Yes | Yes | N/A | Yes | Yes | N/A | N/A | N/A |
| Jang et al., 2016 | Yes | Yes | N/A | Unclear | Yes | Yes | Yes | Yes | N/A |
| Gao et al., 20222 | Yes | Yes | Yes | Yes | Yes | No | Unclear | Unclear | Yes |
| Maller et al., 2014 | Yes | Yes | Yes | Yes | Yes | Yes | Yes | Yes | Yes |
| Maller et al., 2014 | Yes | Yes | Yes | Yes | Yes | Yes | Yes | Yes | Yes |
| Simos et al., 2023 | Yes | Yes | Yes | Yes | Yes | Yes | Yes | Yes | Yes |
| Luo et al., 2023 | Yes | Yes | Yes | Yes | Yes | Yes | Yes | Yes | Yes |
| Q1: Were the groups comparable other than the presence of disease in cases or the absence of disease in controls? Q2: Were cases and controls matched appropriately? Q3: Were the same criteria used for identification of cases and controls? Q4: Was exposure measured in a standard, valid and reliable way? Q5: Was exposure measured in the same way for cases and controls? Q6: Were confounding factors identified? Q7: Were strategies to deal with confounding factors stated? Q8: Were outcomes assessed in a standard, valid and reliable way for cases and controls? Q9: Was the exposure period of interest long enough to be meaningful? Q10: Was appropriate statistical analysis used? | | | | | | | | | |
